# Consensus quality indicators for monitoring multiple sclerosis

**DOI:** 10.1016/j.lanepe.2024.100891

**Published:** 2024-03-29

**Authors:** Isabel Voigt, Stefanie Fischer, Undine Proschmann, Urszula Konofalska, Peggy Richter, Hannes Schlieter, Thomas Berger, Sven G. Meuth, Hans-Peter Hartung, Katja Akgün, Tjalf Ziemssen

**Affiliations:** a Center of Clinical Neuroscience, Department of Neurology, Medical Faculty and University Hospital Carl Gustav Carus, TUD Dresden University of Technology, Fetscherstraße 74, Dresden 01307, Germany; b Research Group Digital Health, Faculty of Business and Economics, TUD Dresden University of Technology, Dresden 01062, Germany; c Department of Neurology, Medical University of Vienna, Währinger Gürtel 18-20, Vienna 1090, Austria; d Comprehensive Center for Clinical Neurosciences & Mental Health, Medical University of Vienna, Währinger Gürtel 18-20, Vienna 1090, Austria; e Department of Neurology, Medical Faculty and University Hospital Düsseldorf, Heinrich-Heine-University Düsseldorf, Moorenstr. 5, Düsseldorf 40225, Germany

**Keywords:** Disease management, Monitoring, Multiple sclerosis, Quality indicators, Quality management, Quality of care

## Abstract

Multiple sclerosis (MS) as a chronic, degenerative autoimmune disease of the central nervous system has a longitudinal and heterogeneous course with increasing treatment options and risk profiles requiring constant monitoring of a growing number of parameters. Despite treatment guidelines, there is a lack of strategic and individualised monitoring pathways, including respective quality indicators (QIs). To address this, we systematically developed transparent, traceable, and measurable QIs for MS monitoring. Through literature review, expert discussions, and consensus-building, existing QIs were identified and refined. In a two-stage online Delphi process involving MS specialists (on average 53 years old and with 25 years of professional experience), the QIs were evaluated for content, clarity, and intelligibility, resulting in a set of 24 QIs and checklists to assess the quality of care. The final QIs provide a structured approach to document, monitor, and enhance the quality of care for people with MS across their treatment journey.

## Introduction

Measuring the quality of care is of extraordinary interest when it comes to chronic diseases like multiple sclerosis (MS) that require lifelong treatment with constant adjustments due to its high complexity. Quality, as defined by the United States Institute of Medicine, is “the degree to which health services for individuals and populations increase the likelihood of desired health outcomes and are consistent with current professional knowledge”. In addition, the Institute defines the general aims “that care should be safe, effective, patient-centered, timely, efficient, and equitable”.^1^ Quality indicators (QIs) may help measure and improve these aims by assessing and monitoring the care processes. QIs represent valid and reliable tools for the evaluation of healthcare quality. Their purpose is to compare actual patient care to ideal criteria. QIs are constructed using guidelines, evidence-based medicine, and best practice consensus.^2^, ^3^, ^4^, ^5^, ^6^, ^7^

In managing MS with its longitudinal and heterogeneous course, large amounts of data are generated from different processes with numerous parameters, assessment tools, interventions, and effects. Consequently, a sage and structured approach is required to conceptualize a high-quality and personalized management of the disease. Several guidelines describe pharmacological therapies for MS in detail.^8^, ^9^, ^10^, ^11^ The importance of an early treatment start respective timely treatment optimization in routine clinical treatment of MS is pointed out by numerous authors with reference to various studies and the comprehensive data on the significance of, e.g., relapses, early changes on Expanded Disability Status Scale (EDSS), and the role of MRI.^12^, ^13^, ^14^ Special efforts to establish time-based consensus standards in MS treatment have been undertaken by a working group of the international Brain Health Initiative.^15^ However, there is a lack of strategic and individualized treatment concepts, especially of respective QIs, which are the prerequisites for high-quality and personalized MS management.

For this reason, the research team aimed to develop transparent, traceable, and measurable QIs for the monitoring process in MS management based on existing guidelines and recommendations and in consensus with MS experts. Monitoring can be considered as two pathways, one to monitor disease activity and progression and the other to monitor interventions.^16^, ^17^, ^18^ This paper presents the development of QI to monitor MS disease activity and progression.

## Methods

Currently, there is no consensus on which methodological approach is best to develop QIs.^3^ However, there is a strong tendency that the most promising approach seems to be to use a deductive and an inductive approach jointly. That means QIs should also be derived from the best available scientific evidence and existing data and its variations.^19^ Additionally, QIs should be meaningful (audience(s) will find the information produced useful for a purpose), scientifically acceptable (measure will produce consistent and credible results), feasible (it can be implemented), and useable (target audience can understand the results and use them for decision making).^20^, ^21^, ^22^

**Fig. 1:**
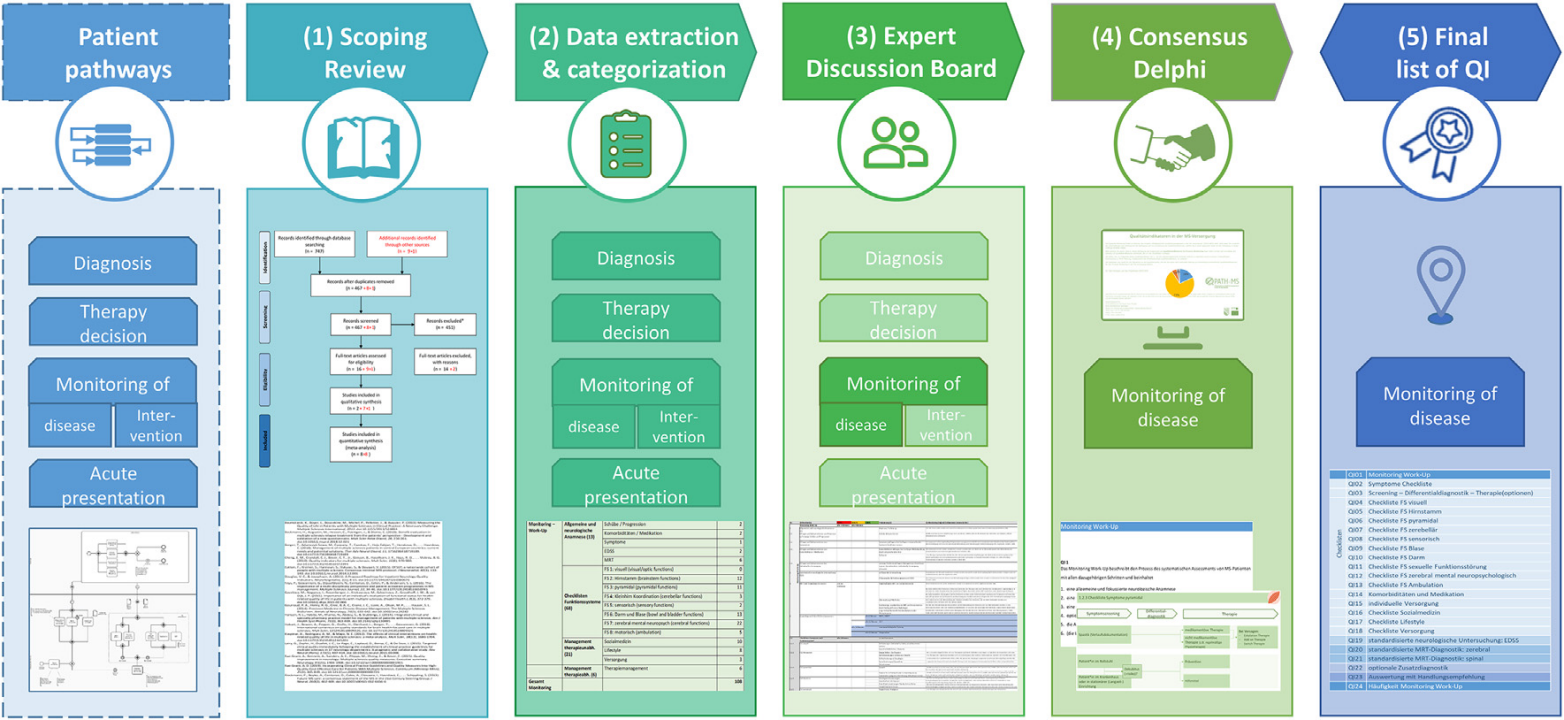
Development of QI for disease monitoring.

The research team applied a combination of literature study, expert discussion, and consensus-building for developing QIs for the pathway of disease activity and progression monitoring (hereafter, for readability reasons, referred to as disease monitoring) and applied evaluation criteria to reach the best results. The development consists of five steps: (1) Scoping review of literature and guidelines, (2) Extraction and categorization of QIs and recommendations from literature and guidelines, (3) Expert discussion board with researchers and neurologists experienced in MS for the transformation of QIs and recommendations into a set of QIs, (4) Two online consensus rounds with a panel of neurologists experienced in MS, and (5) Finalization of the set of QIs (Fig. 1). The research team was composed of research associates and neurologists experienced in MS.

### Scoping review

To review the literature and available evidence on QIs in MS management and potential gaps in MS quality measurement in general, the research team performed a scoping review according to the research question: What QIs are available for the management of MS and the care of people with MS (pwMS)? The aim was to identify relevant studies that examined existing QIs and QI sets for MS. Although the focus was on the monitoring process, the research initially included QIs and QI sets for overall MS management.^23^, ^24^, ^25^, ^26^ The scoping review was conducted based on literature guidance.^24^^,^^26^

**Search strategy**

The following search algorithms were used for systematic search in the PubMed and Web of Science databases. For PubMed, we used the search string:

((“Multiple sclerosis” [MeSH Terms]) OR (“Multiple Sclerosis” [Title/Abstract]) AND (“care” [Title/Abstract]) AND (((“Quality” [Title/Abstract] OR “Outcome∗” [Title/Abstract]) AND (“Indicator∗” [Title/Abstract] OR “Measure∗” [Title/Abstract] OR “Standard∗” [Title/Abstract])) OR (“quality indicators, health care” [MeSH Terms]))) AND ((humans [Filter]) AND (2010/1/1:3000/12/12 [pdat]) AND (english [Filter] OR german [Filter])).

For Medline, we used the search string:

(AB = (Multiple Sclerosis AND care) AND AB = ((quality OR outcome∗) AND (indicator∗ OR measure∗ OR standard∗))) AND LANGUAGE: (German) AND (English) Indexes = SCI-EXPANDED, SSCI, A&HCI, CPCI-SSH, BKI-S, BKI-SSH, ESCI, CCR-EXPANDED, IC Timespan = 2010–2021.

The searches were carried out in January 2021. All types of study designs were included. Only studies published in the last ten years (2011–2021) were deemed eligible. Restriction to German and English language was applied.

#### Study selection and eligibility criteria

The review consisted of a multi-step approach, including title and abstract screening and full-text assessment. Studies that did not focus on QIs for MS care were not included. Duplicate articles found when searching two databases were filtered out. Two reviewers independently selected articles identified through the search algorithms by analyzing titles and abstracts. Articles deemed relevant by the reviewers and met the inclusion criteria (Table 1) were included in a full-text review (Table 2, Table 3).^8^^,^^9^^,^^12^^,^^15^^,^^27^, ^28^, ^29^, ^30^, ^31^, ^32^, ^33^, ^34^, ^35^, ^36^, ^37^, ^38^, ^39^, ^40^, ^41^, ^42^, ^43^, ^44^, ^45^, ^46^ Disagreement concerning full-text articles was resolved through discussion with two more reviewers until a complete consensus was reached. The reviewers hold several meetings to discuss challenges and ambiguities related to study selection. Additionally, the research team decided to include gray literature, such as guidelines and recommendations, identified through expert knowledge and a supplementary internet search. Fig. 2 shows the flowchart of the study selection process. A paper that appeared after the database search in the summer of 2021 was also considered.^5^

**Table 1:** Inclusion and exclusion criteria for study selection.

Inclusion criteria	Exclusion criteria
All articles, that focus on (the generation of) QI (sets) for MS management and care, e.g., - Quality indicators/indicator sets, - Quality improvement - (Quality) guidelines/standards (both in general and for symptoms) - Inclusion of validated and non-validated QI (due to rarely existing validated QI)	All articles dealing with - Diagnosis, e.g., MRI, imaging, spinal cord, biomarker, ophthalmology - Therapies, e.g., disease-modifying therapy, cannabinoids, diet, physical activity - Validation of functional tests - Assessment tools (but not quality) - Influencing factors of MS - Costs, efficacy, safety, adherence - PRO(M), PRE(M) - Symptoms - Other topic than QI in MS

**Table 2:** Selected reports identified for full-text review through database searching.

#	Author	Year	Title	Included
1	Baumstarck et al.	2013	Measuring the Quality of Life in Patients with Multiple Sclerosis in Clinical Practice: A Necessary Challenge	no
2	Beckmann et al.	2019	Benefit evaluation in multiple sclerosis relapse treatment from the patients’ perspective–Development and validation of a new questionnaire	no
3	Berger et al.	2018	Management of multiple sclerosis patients in central European countries: current needs and potential solutions	no
4	Cheng et al.	2010	Quality indicators for multiple sclerosis	yes
5	Cotton et al.	2015	OFSEP, a nationwide cohort of people with multiple sclerosis: Consensus minimal MRI protocol	no
6	Douglas et al.	2011	A Proposed Roadmap for Inpatient Neurology Quality Indicators	no
7	Feys et al.	2016	The importance of a multi-disciplinary perspective and patient activation programmes in MS management	no
8	Gavelova et al.	2015	Importance of an individual’s evaluation of functional status for health-related quality of life in patients with multiple sclerosis	no
9	Gourraud et al.	2014	Precision Medicine in Chronic Disease Management: The Multiple Sclerosis BioScreen	no
10	Hanson et al.	2014	Integrated clinical and specialty pharmacy practice model for management of patients with multiple sclerosis	no
11	Hobart et al.	2019	International consensus on quality standards for brain health-focused care in multiple sclerosis	yes
12	Kuspinar et al.	2012	The effects of clinical interventions on health-related quality of life in multiple sclerosis: a meta-analysis	no
13	Lairy et al.	2015	Targeted clinical audits immediately following the establishment of clinical practice guidelines for multiple sclerosis in 17 neurology departments: A pragmatic and collaborative study	no
14	Rae-Grant et al.	2019	Incorporating Clinical Practice Guidelines and Quality Measures Into High-Quality Cost-Effective Care for Patients With Multiple Sclerosis	no
15	Rieckmann et al.	2013	Future MS care: a consensus statement of the MS in the 21st Century Steering Group	no

**Table 3:** Selected additional reports for full-text review identified through other sources.

#	Short title	Year	Title	Included
1	NICE-PE	2012	NICE. Patient experience in adult NHS services. Quality standard	no
2	NICE-MS	2016	NICE. Multiple sclerosis. Quality standard	yes
3	EAN	2018	ECTRIMS/EAN Guideline on the pharmacological treatment of people with multiple sclerosis	yes
4	AAN	2015,2021	Quality improvement in neurology: Multiple sclerosis quality measures: Executive summary. Quality Improvement in Neurology. Multiple Sclerosis Quality Measurement Set 2020 Update	yes
5	DGN	2021, 2023	Diagnose und Therapie der Multiplen Sklerose, Neuromyelitis-optica-Spektrum-Erkrankungen und MOG-IgG-assoziierten Erkrankungen, S2k-Leitlinie.	yes
6	MAGNIMS	2021	2021 MAGNIMS–CMSC–NAIMS consensus recommendations on the use of MRI in patients with multiple sclerosis	yes
7	MSTCG	2021	Multiple Sclerosis Therapy Consensus Group (MSTCG): position statement on disease-modifying therapies for multiple sclerosis (white paper)	no
8	Drug-specific: KKNMS	Up-to-date	Qualitätshandbuch MS/NMOSD. Empfehlungen zur Therapie der Multiplen Sklerose/Neuromyelitis-optica-Spektrum-Erkrankungen für ÄrztInnen (Webversion)	yes
9	Drug-specific: pharmacological instructions	Up-to-date	Several pharmacological instructions for DMTs (search for Product Information for DMTs for MS on https://www.ema.europa.eu/en/medicines)	yes

**Fig. 2:**
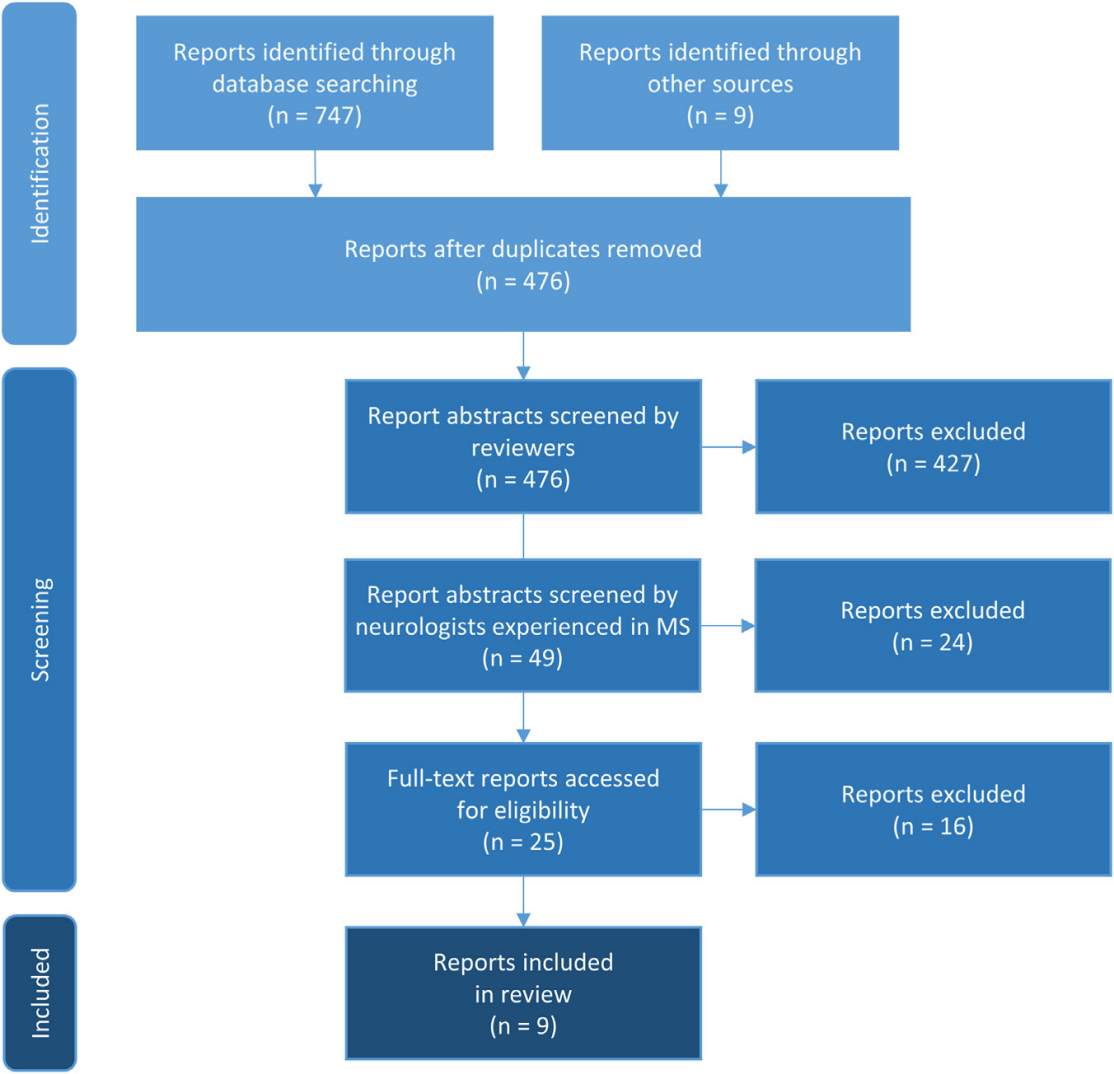
Report of searching results based on PRISMA.^47^

### Data extraction and categorization

The research team developed a data collection sheet to confirm the studies’ relevance and to extract all QIs. They also predefined categories and characteristics to which the QIs could be assigned. Two reviewers collected references and the original full text of the extracted potential QIs or recommendations and categorized them in terms of (1) Donabedian’s classification of health care quality (structure, process, outcome), (2) the core process of MS management (diagnosis, therapy decision, monitoring, acute presentation), (3) if applicable, the characteristics of QI (effectiveness and safety, patient-centeredness, and continuity), and, (4) if the QI refers to a symptom, the respective functional system according to EDSS (visual, brainstem, pyramidal, cerebellar, sensory, bowel and bladder, cerebral/mental/neuropsychological, ambulation and mobility).^2^^,^^14^^,^^48^ Where appropriate, they marked them with the suitable exclusion criterion, i.e., QI is not workable/measurable, QI gives no hint for improvement potential, QI lacks comprehensibility and/or efforts for data acquisition are above benefits, QI is a duplicate/not adaptable. Four other neurologists experienced in MS also rated the listed QIs by category or exclusion criteria, each for himself, without seeing the results of the others.

### Expert discussion board

The expert discussion board consisted of one research associate and four neurologists experienced in MS. In several meetings, they discussed the procedure for sorting and potentially revising, collating, and reformulating the QIs. Finally, they compiled a set of QIs for evaluation by two consensus rounds with an MS expert panel via an online survey.

### Expert panel consensus

#### Modified delphi process

To evaluate the developed QIs, the research team conducted a two-round survey based on Delphi methodology to find consensus on the collated QIs with the additional aim of improving, completing, and prioritizing the QIs.^48^, ^49^, ^50^ To this end, the panel of MS experts was asked to evaluate each QI using predetermined criteria and to comment on each QI in a free text section. The advantage of the Delphi process is that involved experts often have access to information about a topic that is more up-to-date than what can be found in the extant literature and that group decisions are more reliable than decisions made by a single person.

#### Evaluation criteria

The criteria for evaluating QIs should help to assess them for relevance (e.g., importance and usefulness for care), scientific criteria (e.g., validity, reliability, clarity), and practicality (e.g., interpretability for patients).^19^, ^20^, ^21^^,^^51^, ^52^, ^53^ Based on this, the research team designed three criteria for evaluating the QIs for disease monitoring regarding the questions (1) Does the QI make sense? (Yes/No), (2) Does the QI meet a pivotal issue of MS care? (The QI is essential/desirable/not important for MS care) and (3) Does this QI matter to MS patients in the sense of patient empowerment, i.e., should this QI be communicated to the patient? (Yes/No).

#### Selection of panel experts

Experts were selected based on research, implementation of projects, and professional experience with MS. They were personally invited by e-mail and got a personalized link to conduct the survey. Reminders were sent if necessary. All the experts received information about the aim of the study, the modified Delphi process, and instructions to rate the QIs. For the first evaluation round (July–September 2022), 123 experts were contacted, 62 of whom participated and 55 of whom also provided demographic data. In the second round (December 2022–January 2023), 55 of the 122 experts contacted participated, 49 of whom also provided demographic data. Of the participants in the first round, 35 also took part in the second round.

#### Online survey

The QIs were formatted into a clear and understandable form to display in an online survey using Lime Survey software (version 5.5.0 + 221,219). For the first round, the survey consisted of the QIs to be evaluated according to the specified criteria, free text fields, and a request for demographic data. For the second round, the revised QIs were presented with the aggregated evaluation results from the first round with clearly marked revisions. At the end of round two, the experts were asked to prioritize the five most essential QIs.

#### Analysis

The data was anonymized for the analysis. For both rounds, the researchers conducted a descriptive analysis (mean, modus, median, range) of demographic data and consent on the QIs using Microsoft Excel. All QIs with an agreement (“yes” for criteria 1 and 3) of more than 75% and with a rating of “essential” or “desirable” (for criterion 2) were accepted as eligible for the final QI set. The free text comments were abstracted, merged, and categorized regarding clarity, comprehensibility, content, feasibility, and communication with or to patients. Results of the first round were presented to interested panel members in an online presentation and discussion round. Based on this, QIs were supplemented, expanded, or modified to show them in the second round, together with the aggregated results of the first round. The free text comments from the second round were also processed and incorporated into the existing list of QIs, resulting in a final list of QIs.

## Results

### Data extraction and categorization

**Fig. 3:**
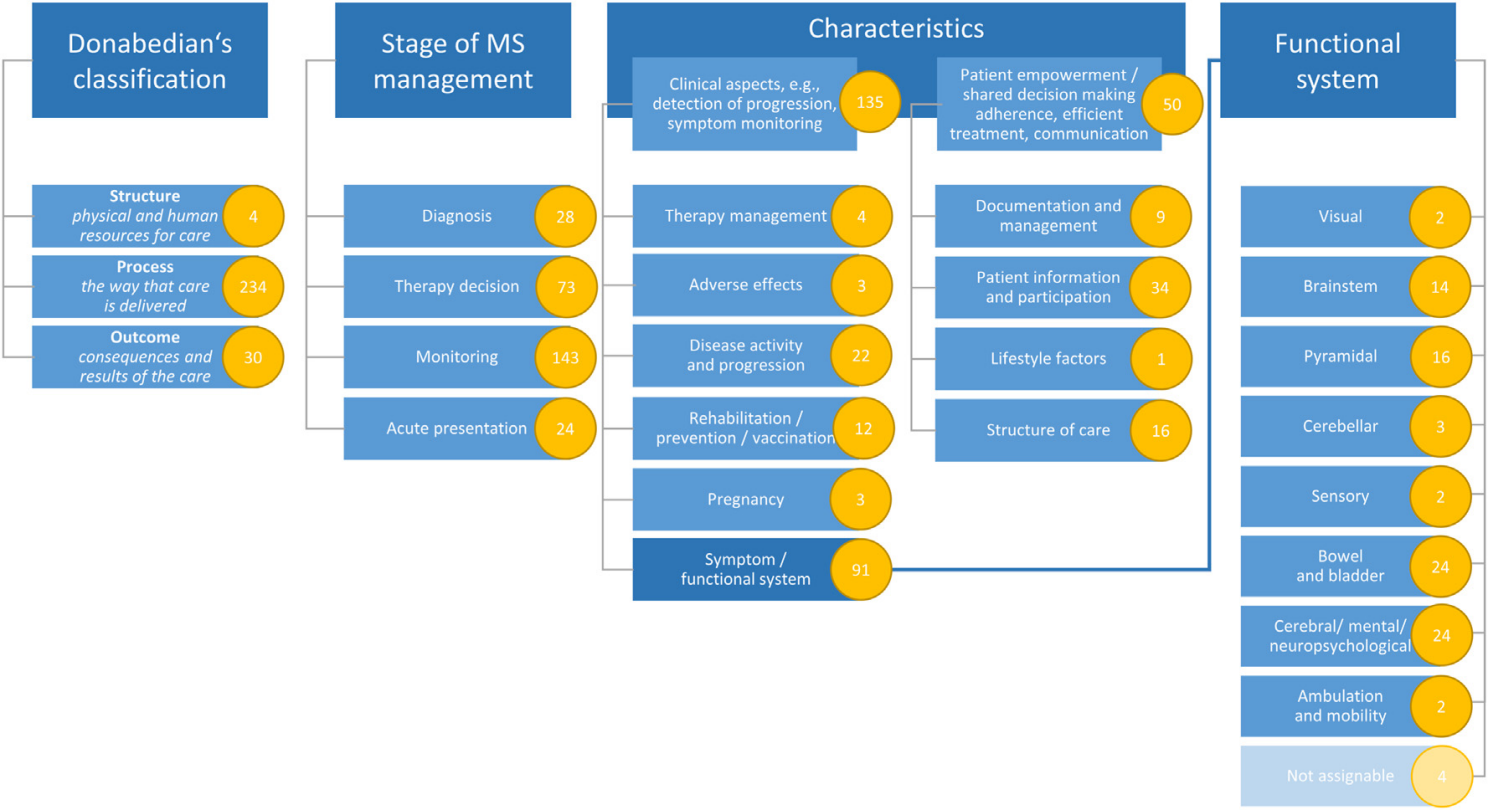
First categorization of 268 potential non-drug-specific QIs.

From the 9 reports selected through the scoping review, the research team transferred 883 potential QIs and recommendations to the data collection sheet and assigned them to the predefined categories and characteristics (Fig. 3) or to the corresponding exclusion criterion. As 615 potential QIs out of two reports (# 8 and 9 in Table 3) were drug-specific recommendations or instructions for intervention monitoring which refer to the drugs alemtuzumab, cladribine, dimethyl fumarate, fingolimod, glatiramer acetate, interferon, natalizumab, ocrelizumab, and teriflunomide, they were not included further in the analysis, as the focus is on disease monitoring. The other 268 potential QIs were non-drug-specific QIs for disease monitoring which were discussed further by the expert discussion board.

### Expert discussion board

After QI extraction and categorization, members of the expert discussion board discussed and re-sorted QIs, merged similar QIs, and excluded duplicates. A total of 154 potential QIs remained, of which 110 QIs were for monitoring, divided into four structural, 95 process, and 11 outcome QIs according to the Donabedian classification.

**Fig. 4:**
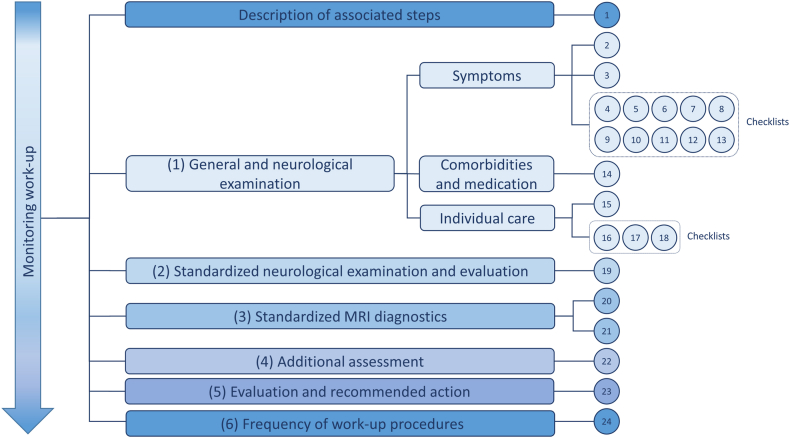
Expert categorization of QIs.

In the next step, experts agreed on a standardized procedure for the monitoring process (monitoring work-up).^16^ They assigned the potential monitoring QIs to meaningful steps (Fig. 4) within the disease monitoring process: (1) General and neurological history, including checklists for symptoms, medication and comorbidities, and individual care, (2) Standardized neurological examination and evaluation, (3) Imaging, (4) Additional assessment, (5) Evaluation and interaction with the patient, and (6) Frequency of work-up procedures. During the assignment, the QIs were also revised, partially reformulated, and expanded where necessary. The expert discussion board finally compiled a set of 24 QIs, including several checklists for the monitoring process (Supplemental material #1). The QIs were then compiled for evaluation by two expert consensus rounds via an online survey. The final QIs are presented in Table 4.

**Table 4:** Final list of QIs.

(Sub)group	#	QI in detail	References
General and neurological history			
Monitoring Work-Up	**QI01**	The monitoring Work-Up describes the process of a systematic longitudinal assessment of MS patients with all associated steps and includes 1. A general and focused neurological history 2. A standardized neurological examination and evaluation 3. Standardized MRI diagnostics 4. Optional additional assessments 5. The evaluation of 1.-4. With recommendation for action (recommendation for action means: a concrete proposal for further drug and/or non-drug treatment is derived from the thorough synopsis of all parameters evaluated in 1–4 (see also QI 23) 6. (the respective implementation of the monitoring by physician/patient)	Expert discussion board
Symptoms	**QI02**	The general and focused neurological history includes asking for and documenting symptoms. The questioning is done by means of checklists for each functional system based on the EDSS (checklists for symptoms in functional systems visual, brainstem, pyramidal, cerebellar, sensory, bladder, bowel, sexual dysfunction, cerebral mental neuropsychological, ambulation and mobility).	DGN
	**QI03**	Within the functional systems, the query of symptoms follows the scheme Symptom Screening → Differential Diagnosis → Action/Therapy (options).	Expert discussion board
	QI04^a^	Checklist for symptoms in functional system visual	
	QI05^a^	Checklist for symptoms in functional system brainstem	Cheng, DGN
	QI06^a^	Checklist for symptoms in functional system pyramidal	Cheng, DGN
	QI07^a^	Checklist for symptoms in functional system cerebellar	DGN
	QI08^a^	Checklist for symptoms in functional system sensory	DGN
	QI09^a^	Checklist for symptoms in functional system bladder	DGN
	QI10^a^	Checklist for symptoms in functional system bowel	Cheng, DGN
	QI11^a^	Checklist for symptoms in functional system sexual dysfunction	Cheng, DGN
	QI12^a^	Checklist for symptoms in functional system cerebral mental neuropsychological	Cheng, DGN, AAN, Hobart
	QI13^a^	Checklist for symptoms in functional system ambulation and mobility	Cheng, DGN
Comorbidities and medication	**QI14**	The general and focused neurologic history includes inquiring about and documenting comorbidities and medications. Inquiring is done using a therapy-dependent management checklist: 1. Query comorbidities with their therapies. 2. Status query DMT, symptomatic therapy(s), adjuvants, complementary/alternative therapies, 3. Determination/review/change of therapy goals 4. Communication of therapy goals/management/risks to patient 5. Therapy-specific measures: Assessment DMT and medication specific management as part of process therapy	DGN, Hobart, Cheng, pharmacological instructions, KKNMS
Individual care	**QI15**	The general and focused neurological history also includes inquiring about and documenting the individual care situation. The inquiries are made using checklists for therapy-independent management.	Expert discussion board
	QI16	The social medicine checklist includes: - Preventive medical checkups + vaccinations according to local recommendations are queried and documented. - Degree of care, degree of disability, reduction in earning capacity, ability to work, family care, ongoing social medical procedures/appeals are queried and documented - Self-help options are pointed out	Cheng, DGN
	QI17	For the lifestyle checklist, the following items are queried and documented: - Ability to perform activities of daily living (ADL) - Problems at work and occupation - Quality of life - Lifestyle habits (sports, exercise, diet, sleep, relaxation, cardiovascular risk factors) - Pregnancy (DMT adjustment if necessary) - Participation (social life, art, culture)	Cheng, AAN, EAN, Hobart
	**QI18**	For the care checklist, it is asked and documented whether the patient has access to - MS care (neurologist, MS specialist) - Primary care (family doctor or similar) - Nursing support/assistance, care - Rehabilitation if indicated, preferably in an MS-experienced rehabilitation facility, if necessary in a MS-specialized clinic - Palliative care with indication	Cheng, NICE-MS, DGN
Standardized neurological examination and evaluation	**QI19**	Standardized neurological examination and evaluation is performed by conducting the EDSS neurological examination according to functional systems: visual, brainstem, pyramidal, cerebellar, sensory, bowel and bladder, cerebral mental neuropsychological, ambulation and mobility.	AAN, NICE-MS
Standardized MRI diagnostics	**QI20**	Cerebral MRI is performed regularly and images and reporting should be performed according to MAGNIMS protocol: - Mostly without contrast agent for follow-up examinations - Measurement of new or clearly enlarging T2 lesions - depending on PML risk (low: 1x yearly, high: 2–4x yearly) - Interpretation by radiologists with MS expertise - Comparison with reference MRI	Hobart, DGN, EAN
	**QI21**	Spinal MRI is important for diagnosis and for assessing the initial extent of CNS involvement (i.e., disease burden), Imaging and reporting should be performed according to MAGNIMS protocol: - Detection of new or clearly enlarging T2 lesions - Exclusion of possible comorbidity with spine or spinal cord involvement - Interpretation by radiologists with MS expertise Spinal MRI is not recommended as a routine monitoring procedure, but may be useful in patients whose clinical progression cannot be explained by brain MRI findings, or in pending treatment change decisions.	MAGNIMS
Additional assessments	QI22	Optionally, or if there are indications in the medical history of an acute worsening of the symptoms in the sense of relapses/disease progression, additional diagnostics with the performance of: - Standardized functional testing with MSFC/MSPT every 6 months - Gait analysis (e.g. with T25FW, MSWS, MSSS-88) every 12 months - OCT every 12 months - Neuropsychological testing every 12 months The elicitation should be carried out with quantitative methods so that an objective comparison of previous findings/initial values with follow-up values of the surveyed parameters is possible (assessment of progression).	Expert discussion board
Evaluation with recommended action	**QI23**	The evaluation and communication of the monitoring work-up includes - The detection and documentation of clinical or paraclinical signs of disease activity, worsening of MS symptoms, disease progression, relevant concomitant factors (e.g. comorbidities) - Communication of these results to the patient - The development of a recommendation for action in communication with the patient - (The implementation of the recommended action(s) by HCP/patient)	Cheng
Frequency	QI24	A monitoring work-up MUST be performed every 12 months/SHOULD be performed every 6 months and as needed (relapse or symptom worsening), depending on the course of the disease and the patient’s circumstances.	Hobart

a See Fig. 5 for details | QIs ranked to be priority by the expert panel are marked in bold.

### Expert panel consensus

The characteristics of the panel experts were very similar in both rounds. The participating experts were mainly from Germany, with a few from Austria and Switzerland. They were, on average, 53 years old and had 25 years of professional experience, 19 of which specialized in MS (Supplemental material #2).

Overall, the evaluation process revealed a high degree of agreement with the monitoring work-up procedure. The agreement with criterion (1) “Does the QI make sense?” was very high for all QIs (with an average of 88% of experts), albeit with a slight increase in round 2 (97%). Criterion (2) “Does the QI meet a pivotal issue of MS care?” was mostly answered with “it is essential” or “it is desirable for MS care”, with slight shifts between “necessary” and “desirable” from round 1 to round 2. For criterion (3) “Does this QI matter to MS patients in the sense of patient empowerment, i.e., should this QI be communicated to the patient?” overall agreement was not as high as for criterion (1). However, on average, 79% of experts in the first round and 82% of experts in the second round still agreed that they should talk to the patient about the objectives of the respective QI (Supplemental material #3). From the free-text comments, it can be concluded that the experts have a great desire for standardization of processes and examinations. In the first round, some of the experts on the panel had relatively many comments on clarity and comprehensibility, content, practicability, and communication with or to the patient. In the second round, the number of comments was vastly lower than in the first round. However, the experts annotated some QIs and suggested adding some details to certain QIs. Suggestions were largely considered and included in the QI set. The difficulty of realizing the aspirations of some QIs remained due to the continuing lack of capacities.

### Final list of QIs

The final list of 24 QIs is presented in Table 4 with related references. QI01 was drafted by the expert discussion board and describes the associated steps to be fulfilled within the monitoring work-up, defined as a systematic longitudinal assessment of MS patients. This QI summarizes all the steps the neurologist should perform in every monitoring session and enumerates various QIs and recommendations in several references including steps (1) to (5) within the disease monitoring process. In conjunction with the results and parameters of the examinations performed, the neurologist should be able to recommend specific further drug and/or non-drug treatment steps to the patient.

#### General and neurological history, including checklists for symptoms, medication, comorbidities, and individual care (QI02-18)

QI02 refers to a general and neurological history that includes asking about and documenting symptoms, ideally through standardized checklists that focus on the functional systems based on the EDSS.^9^ QI03 was designed by the expert discussion board and specifies the preferred way of asking this question by setting out the steps the neurologist should take. The checklists for symptoms (QI04-13) in the functional systems are detailed in Fig. 5a and b. Of course, symptoms should not only be considered individually and within the respective functional system. Rather, it is particularly important to assess the patient’s situation in the overall view of the general and focused neurological history and other diagnostic and assessment results and to manage it in the best possible way. In addition to the other diagnostic and assessment results, comorbidities and medication (QI14) and the patient’s specific care situation (QI15) should also be queried to complete the overall picture.^8^^,^^9^^,^^15^^,^^30^^,^^42^, ^43^, ^44^^,^^54^ For comorbidities and medication, the expert discussion board drafted a so-called therapy-dependent management checklist with a query of comorbidities with their therapies, status query of disease-modifying therapies (DMTs), symptomatic therapy(s), adjuvants and complementary or alternative therapies, the setting, review or modification of therapy goals, the communication of treatment goals, management and risks to the patient, and for therapy-specific measures an assessment of DMT- and medication-specific management as part of the process therapy.^9^^,^^15^^,^^30^^,^^54^ The expert discussion board created a so-called therapy-independent management checklist for the patient’s specific care situation with QI16, QI17, and QI18. QI16 is a social-medical checklist with queries and documentation of preventive examinations and vaccinations according to local recommendations, degree of care, degree of disability, reduced earning capacity, ability to work, family care, ongoing social-medical procedures or objections, and the recommendation of self-help options.^9^^,^^54^ QI17 is a checklist on lifestyle with questioning and documentation of the ability to carry out activities of daily living (ADL), problems at work and occupation, quality of life, lifestyle habits (sports, exercise, nutrition, sleep, relaxation, cardiovascular risk factors), pregnancy (with DMT adjustment if necessary), and participation in social life, art, and culture.^8^^,^^15^^,^^30^^,^^43^^,^^44^ QI18 is a care checklist that asks and documents whether the patient has access to MS care (neurologist, MS specialist), primary care (family doctor or similar), nursing support, help or care, rehabilitation if indicated (preferably in an MS-experienced rehabilitation facility, if necessary in an MS-specialized clinic), and palliative care if indicated.^9^^,^^30^^,^^42^^,^^44^

**Fig. 5:**
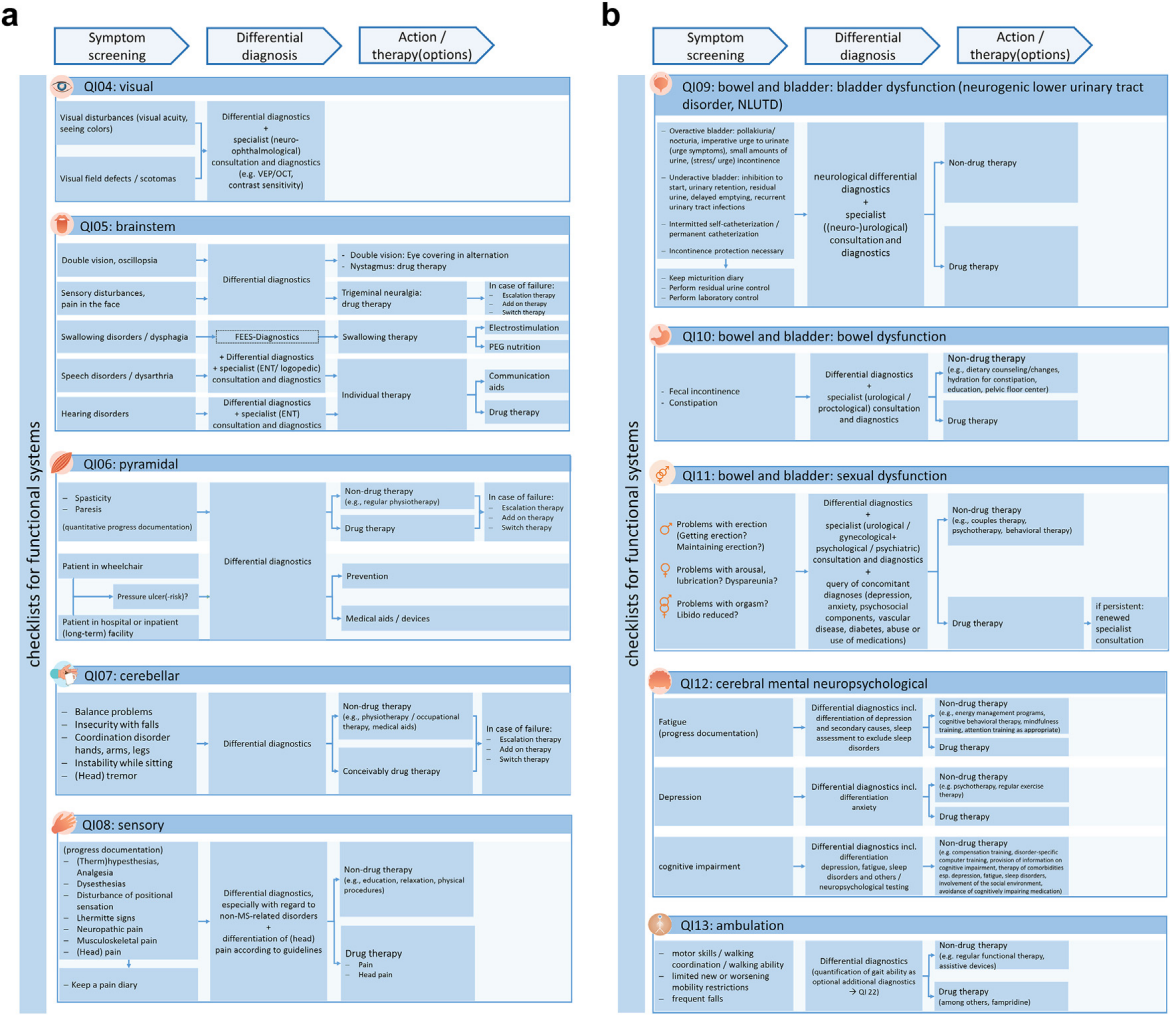
a) Checklists for functional systems “visual”, “brainstem”, “pyramidal”, “cerebellar”, “sensory”. b) Checklists for functional systems “bowel and bladder”, “cerebral mental neuropsychological”, “ambulation”.

#### Standardized neurological examination and evaluation

QI19 prescribes the performance of the EDSS.^43^^,^^44^

#### Imaging

QI20-21 contain recommendations for imaging with a standardized cerebral MRI (QI20) and a standardized spinal MRI (QI21).^8^^,^^9^^,^^15^^,^^46^

#### Additional assessments

QI22 can be performed optionally, or if there are indications in the medical history of an acute worsening of the symptoms in the sense of relapses or disease progression. Additional assessments include standardized functional testing with MS Functional Composite (MSFC) or MS Performance Test (MSPT) every six months, gait analysis with, e.g., timed 25-foot walk (T25FW), 12-item MS walking scale (MSWS) or MS spasticity scale (MSSS-88), every 12 months, optical coherence tomography (OCT) every 12 months, and neuropsychological tests, e.g. with the Brief International Cognitive Assessment for MS (BICAMS) or the Minimal Assessment of Cognitive Function in MS (MACFIMS), every 12 months.^55^, ^56^, ^57^, ^58^, ^59^, ^60^, ^61^, ^62^ To ensure an objective comparison of previous findings or baseline values with follow-up values of parameters, the expert discussion board recommends that the elicitation should be carried out using quantitative methods.

#### Evaluation and interaction with the patient

QI23 covers the comprehensive evaluation of outcomes and interaction with the patient. This includes recognizing and documenting clinical or paraclinical signs of disease activity, worsening MS symptoms, disease progression, and relevant concomitant factors (e.g., comorbidities), communicating these findings to the patient, developing a recommended course of action in communication with the patient, and implementing the recommended action(s) by HCP and patient.^30^

#### Frequency of work-up procedures

QI24 addresses the frequency of work-up procedures in step (6). A monitoring work-up SHALL be performed every 12 months and SHOULD be performed every six months and as needed (relapse or symptom worsening), depending on disease progression and patient.^15^

## Discussion

This paper presents a newly concerted set of 24 QIs for the disease monitoring process in the management of MS. Therefore, the research team first extracted existing QIs and QI sets from the literature, then merged, reduced, re-arranged, and partially expanded them, and finally conducted a two-stage expert survey to assess their content, clarity, and comprehensibility in practice. The final concerted QIs can be used to document, monitor, and ideally improve the quality of the disease monitoring process and, thus, the care of pwMS.

### Strengths and limitations

To our knowledge, this is the first study to create a set of QIs for monitoring based on existing quality recommendations in the literature that is comprehensive, categorized, and reflects the reality of care. In contrast to other disciplines, sufficient and robust quality indicators for MS management are scarce.^5^^,^^63^, ^64^, ^65^, ^66^ Existing indicators only cover segments of MS management (e.g., MS-related symptoms or timelines for specific treatment steps) or do not entirely meet the requirements of importance, scientific validity, feasibility, and usability.^20^, ^21^, ^22^ This is quite surprising considering the importance and necessity of high-quality care for pwMS due to their lifelong burden. Our QI set for the disease monitoring process goes far beyond the existing QIs. The QIs are aligned and provide an overall picture for a complete work-up procedure. Using a combined deductive and inductive approach, we used existing QIs and developed meaningful measures based on areas not previously covered by existing QIs. In several rounds of discussion, our expert discussion board compiled and supplemented the QIs to create a meaningful work-up procedure, including checklists for MS monitoring. By involving a large panel of MS experts in the evaluation of the QIs, there is a high probability that the QIs developed meet the requirements for importance and usability. During the development process, we also took great care to ensure that the QIs meet the requirements of scientific validity by already having a numerator and denominator in mind for each QI. Although there were considerations to integrate numerators and denominators into the survey rounds, we decided to refrain from this in favor of a high participation rate and to concentrate the survey on the content aspects of the QI. It may be that some QIs cannot be recorded well in existing data sources. On the one hand, this can be interpreted as a limitation. On the other hand, we wanted to ensure that all points that are important in monitoring and are significant from a neurologist’s point of view are considered and included. After all, how else can there be further development if we only develop QIs that can be measured with existing data? This does not mean neglecting measurability in practice; it means that the focus has been on the content and that there can be challenges with data availability. This is an opportunity to augment relevant databases. However, we cannot make any reliable statements about the feasibility of QIs, as they have not yet been implemented in practice. Another possible limitation is that despite the search for international QIs and QI sets to cover all relevant aspects, the expert panel consisted only of German-speaking individuals. Most of them practice in a large city and a clinical setting. Only a small number of neurologists in private practices were represented, which may introduce some bias. Thus, the QIs developed are not necessarily universally applicable in all settings, because throughout Germany, there is a strong fragmentation between inpatient hospital care and outpatient care (including primary and specialist care) due to differences in organization and payment. For example, neurologists in private practices may not have sufficient human and technical resources at their disposal. They are glad to be able to provide basic care for the patients and would therefore consider some of the QIs as not feasible. Furthermore, different cultures and different healthcare systems may lead to varying appropriateness of QIs developed by German neurologists in specific settings. While some European countries share similarities with German traditions of medical practice, there are significant differences in diagnosis and clinical management of MS, financial resources available for drug and non-drug therapies, access to and availability of care and medication, and the use of patient registries and databases. Therefore, not all steps of the work-up procedure designed for German neurologists can be implemented in other European countries one-to-one. For example, despite an increase in the number of MRI scanners in Eastern countries, there is still the need for (neuro)radiologists to be educated on MS-related quality criteria.^29^

### Implication for practice and research

Studying the implementation of QIs in different disciplines in practice, a mixed picture emerges. For some disciplines, there are few assessments of the implementation of guidelines or QIs, or they are poorly planned, reported, and measured.^67^ In other disciplines, successful implementation of QIs, at least in the local setting, has been achieved in some cases.^63^^,^^68^, ^69^, ^70^ For our QIs, a future step is to manifest the numerators and denominators and conduct a pilot study to implement QIs in practice. In the pilot and implementation phase, it is crucial to collaborate and build strategic partnerships to engage key stakeholders, involve end users, promote and publicize the QIs, and consider cost-effectiveness and workload. Likewise, it would be helpful to include also pwMS in this phase, which would add their experiential knowledge in refining the QIs. Before and even within a pilot study, another step is to identify the various data sources needed and make them accessible for QI use.^7^^,^^19^^,^^68^^,^^71^^,^^72^ In case the required data is unavailable, databases should be augmented to collect data in a structured, standardized manner and high quality.^73^, ^74^, ^75^, ^76^ The key to successfully implementing QIs follows the PDCA cycle (Plan-Do-Check-Act).^77^ QI development is not a one-time process. It is a continuous cycle involving defining, monitoring, and improving quality.^7^^,^^19^^,^^63^ Therefore, QIs should be regularly reviewed for currency and adjusted as necessary to ensure they reflect the current state of research as much as possible. As a recent study on the association between clinic-level quality of care and patient-level outcomes in MS pointed out, QIs should also be tailored and stratified to patient characteristics like age, sex, symptom constellation, and, above all, disease subtype. In the study, certain QIs correlated with relapse-associated disease subtypes but not with progressive ones.^78^

### Conclusion

The results of our study contribute substantially to a high-quality and personalized management of MS and might be the basis for improving the care for pwMS. Furthermore, the results consider all relevant aspects of the disease and represent a valid and precise contribution to the implementation of high-quality treatment. The conceptualization of the approach was proposed for the first time in literature and can serve as a model for defining treatment QIs in other chronic diseases as well. Our aim is to implement the concerted monitoring of QIs in practical settings in Germany and internationally by involving relevant neurology, legislation, and information technology stakeholders. Integrating the monitoring QIs into patient pathways will enhance the quality of MS management. An improved MS management can increase patient safety, participation, and compliance and pave the way to personalized treatment of pwMS.

**Key messages**

• To date, despite existing treatment guidelines and initiatives on time-based consensus standards for the treatment of people with multiple sclerosis, there have been few sufficient and robust quality indicators for the management of multiple sclerosis.
• This paper presents a newly concerted set of 24 quality indicators for the disease monitoring process in the management of MS, based on existing quality recommendations in the literature that is comprehensive, categorized, and reflects the reality of care.
• To implement quality indicators in practice, it is crucial to collaborate and build strategic partnerships to engage key stakeholders, involve end users and pwMS, promote and publicize the quality indicators, and consider cost-effectiveness and workload.
• The most important task is to identify the various data sources needed and make them accessible for QI use. In case the required data is unavailable, databases should be augmented to collect data in a structured, standardized manner and high quality.
• Quality indicators should be regularly reviewed for currency and adjusted as necessary to ensure they reflect the current state of research as much as possible.

## Contributors

IV: conceptualization, data curation, formal analysis, investigation, methodology, projects administration, writing–original draft, writing–review & editing. SF: data curation, formal analysis, investigation. UP: data curation, formal analysis, investigation. UK: data curation, formal analysis, investigation. PR: investigation, methodology, writing–review & editing. HS: investigation, methodology, writing–review & editing. TB: writing–review & editing. SGM: writing–review & editing. HPH: writing–review & editing. KA: data curation, formal analysis, investigation. TZ: conceptualization, supervision, validation, writing–review & editing.

## Data sharing statement

The datasets generated and/or analyzed during the current study are available from the corresponding author upon reasonable request.

## Declaration of interests

IV, SF, UK, HS, PR, and HPH declare that the research was conducted in the absence of any commercial or financial relationships that could be construed as a potential conflict of interest.

UP received personal consulting fees service from Biogen, Roche and Sanofi and personal payment for Speakers bureaus from Novartis, Merck, Biogen, Bayer and Roche.

TB received unrestricted grants to his institution from Biogen, Bristol-Myers-Squibb, Merck, Novartis, Roche, Sanofi/Genzyme, and TEVA ratiopharm; payments for participation in clinical trials made to his, Institution from Alexion, Bayer, Biogen, Bristol-Myers-Squibb, Merck, Novartis, Octapharma, Roche, Sanofi/Genzyme, and TEVA; personal consulting fees from Almirall, Bionorica, Horizon, Merck, Novartis, Roche, Sandoz, Sanofi; personal payment for lectures, presentations, speakers bureaus, manuscript writing and educational events from Almirall, Bayer, Biogen, Biologix, Bionorica, Bristol-Myers-Squibb, Eisai, GW Pharma, Horizon, Janssen-Cilag, MedDay, Merck, Novartis, Octapharma, Roche, Sandoz, Sanofi/Genzyme, TG Pharmaceuticals, TEVA-ratiopharm and UCB.

SGM receives honoraria for lecturing, and travel expenses for attending meetings from Academy 2, Argenx, Alexion, Almirall, Amicus Therapeutics Germany, Bayer Health Care, Biogen, BioNtech, BMS, Celgene, Datamed, Demecan, Desitin, Diamed, Diaplan, DIU Dresden, DPmed, Gen Medicine and Healthcare products, Genzyme, Hexal AG, Impulze GmbH, Janssen Cilag, KW Medipoint, MedDay Pharmaceuticals, Merck Serono, MICE, Mylan, Neuraxpharm, Neuropoint, Novartis, Novo Nordisk, ONO Pharma, Oxford PharmaGenesis, Roche, Sanofi-Aventis, Springer Medizin Verlag, STADA, Chugai Pharma, QuintilesIMS,Teva, Wings for Life international and Xcenda. His research is funded by the German Ministry for Education and Research (BMBF), Bundesinstitut für Risikobewertung (BfR), Deutsche Forschungsgemeinschaft (DFG), Else Kröner Fresenius Foundation, Gemeinsamer Bundesausschuss (G-BA), German Academic Exchange Service, Hertie Foundation, Interdisciplinary Center for Clinical Studies (IZKF) Muenster, German Foundation Neurology and Alexion, Almirall, Amicus Therapeutics Germany, Biogen, Diamed, DGM e.v., Fresenius Medical Care, Genzyme, Gesellschaft von Freunden und Förderern der Heinrich-Heine-Universität Düsseldorf e.V., HERZ Burgdorf, Merck Serono, Novartis, ONO Pharma, Roche, and Teva.

KA received personal compensation from Roche, Sanofi, Novartis, Merck, Teva, BMS for consulting or speaker service.

TZ received personal research support from Biogen, Novartis, Merck, Sanofi; personal consulting fees from Biogen, Roche, Novartis, Celgene and Merck and personal payment for speakers bureaus from Roche, Novartis, Merck, Sanofi, Celgene, and Biogen.

## Appendix ASupplementary data

#1 QI_First Stage

#2 QI_Demographics

#3 QI_Evaluation
