# Supplementary material for: Consensus quality indicators for monitoring multiple sclerosis
Source: Lancet Reg Health Eur. 2024 Mar 29;40:100891. doi: 10.1016/j.lanepe.2024.100891 (PMC10998202; doi:10.1016/j.lanepe.2024.100891)
Supplement: #1 QI_First Stage [file mmc1.pptx]

## Slide 1
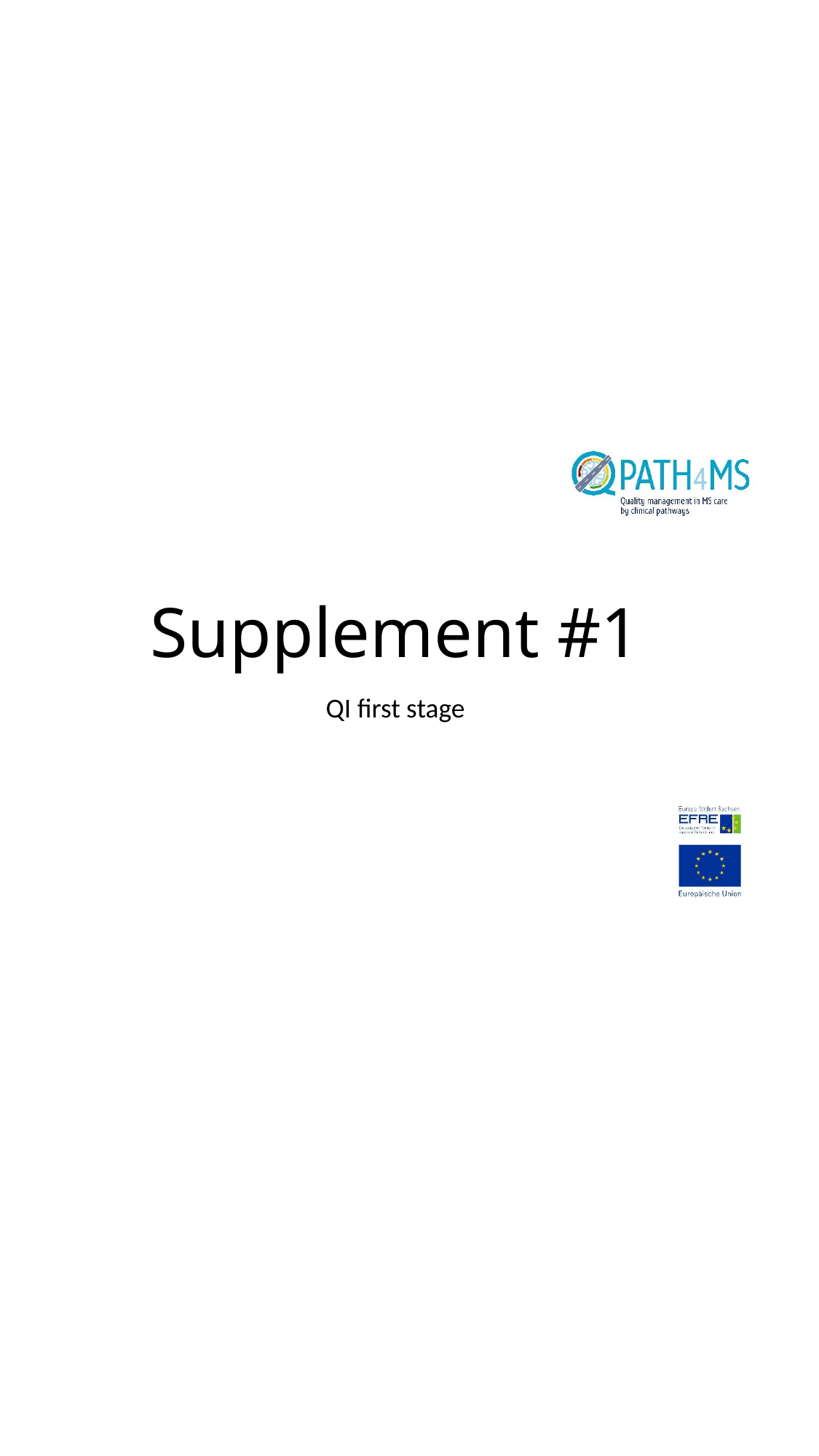

Supplement #1
QI first stage

## Slide 2
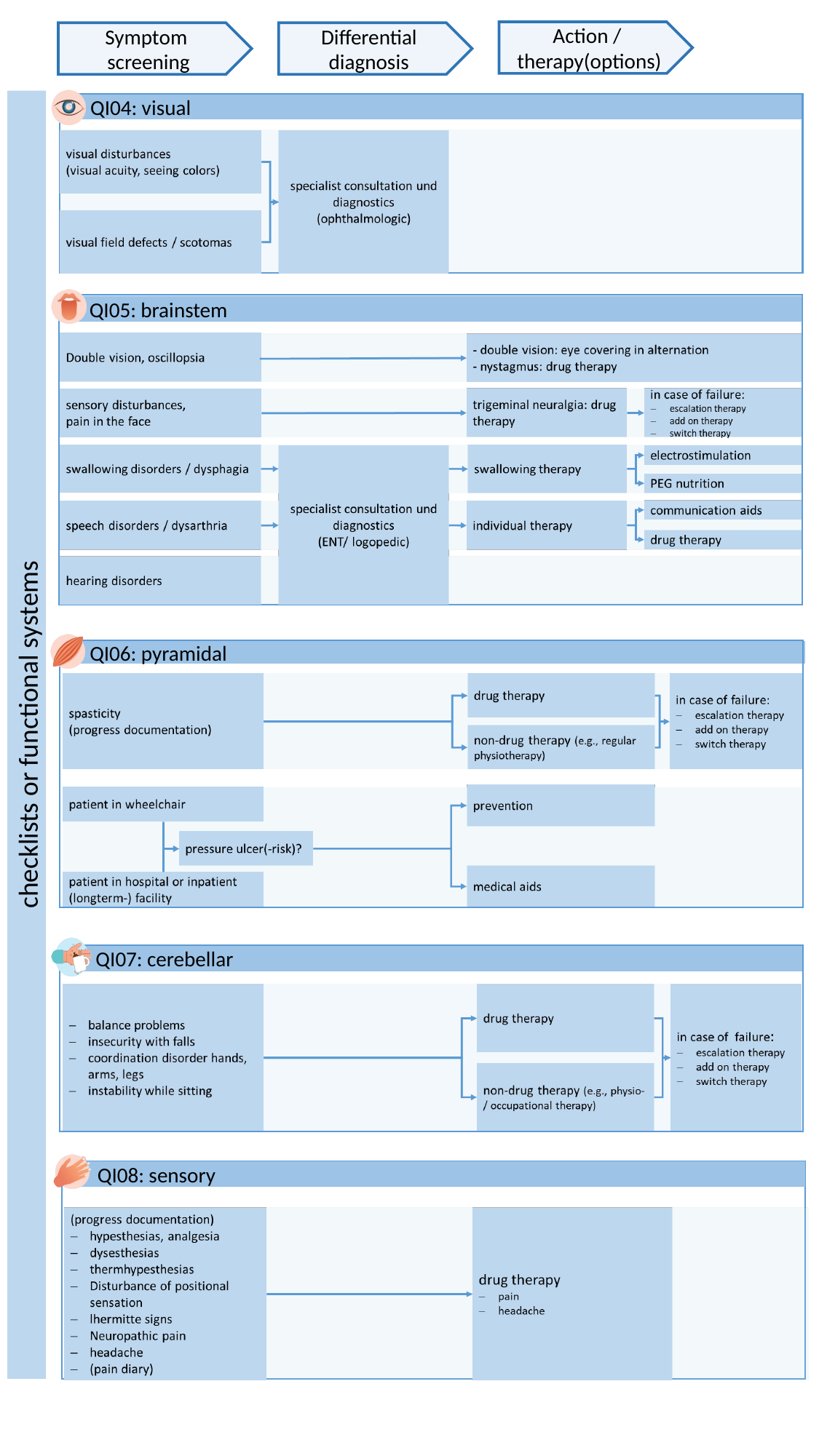

Action /
therapy(options)
Symptom
screening
Differential diagnosis
 QI04: visual
 QI05: brainstem
 QI06: pyramidal
checklists or functional systems
 QI07: cerebellar
 QI08: sensory

## Slide 3
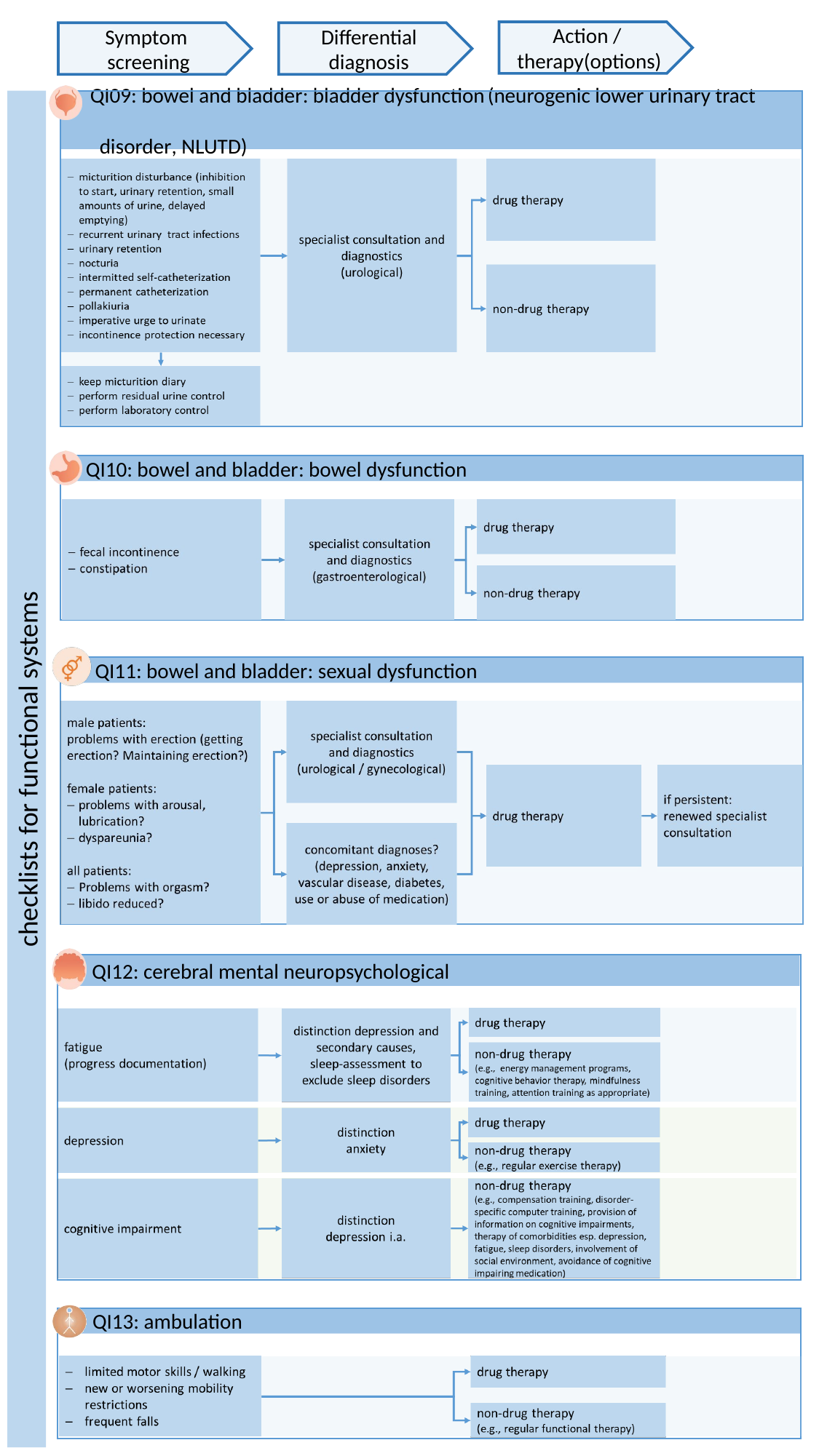

Action /
therapy(options)
Symptom
screening
Differential diagnosis
 QI09: bowel and bladder: bladder dysfunction (neurogenic lower urinary tract
 disorder, NLUTD)
 QI10: bowel and bladder: bowel dysfunction
 QI11: bowel and bladder: sexual dysfunction
checklists for functional systems
 QI12: cerebral mental neuropsychological
 QI13: ambulation
