# Supplementary material for: Consensus quality indicators for monitoring multiple sclerosis
Source: Lancet Reg Health Eur. 2024 Mar 29;40:100891. doi: 10.1016/j.lanepe.2024.100891 (PMC10998202; doi:10.1016/j.lanepe.2024.100891)
Supplement: #2 QI_Demographics [file mmc2.pptx]

## Slide 1
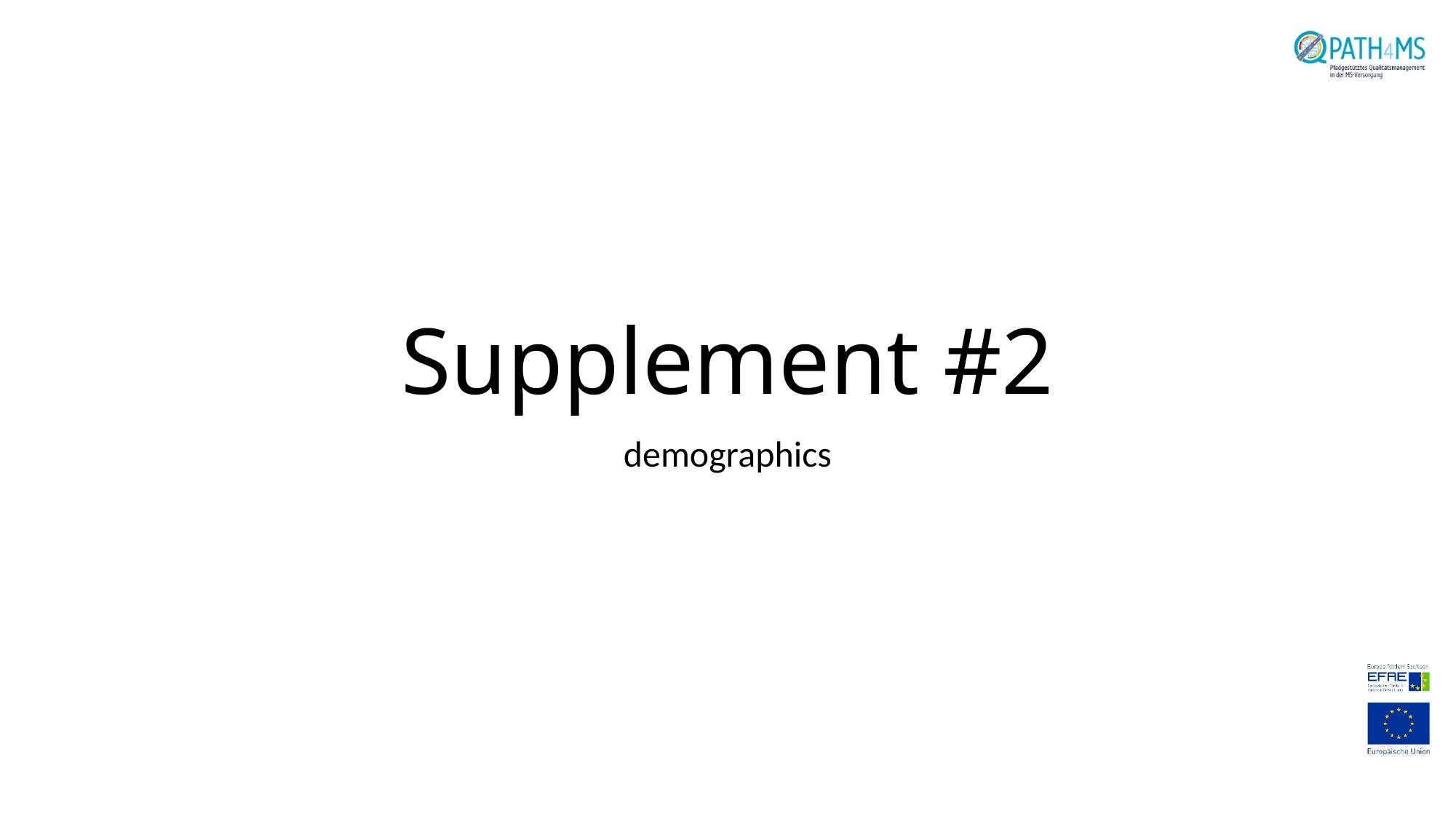

# Supplement #2
demographics

## Slide 2
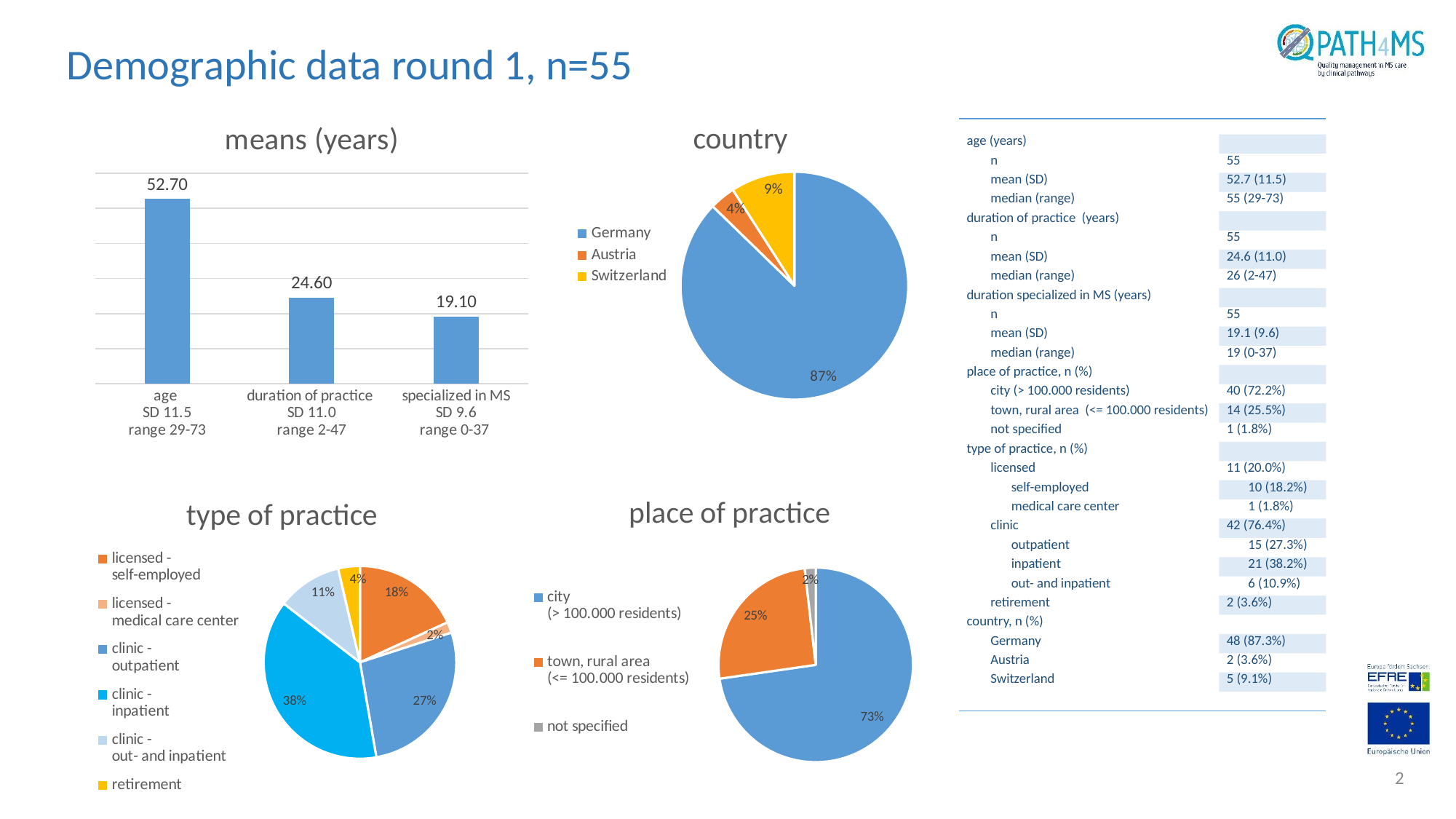

Demographic data round 1, n=55
### Chart: means (years)
| Category | |
|---|---|
| age
SD 11.5
range 29-73 | 52.7 |
| duration of practice
SD 11.0
range 2-47 | 24.6 |
| specialized in MS
SD 9.6
range 0-37 | 19.1 |
### Chart: country
| Category | | |
|---|---|---|
| Germany | 48.0 | 0.8727272727272727 |
| Austria | 2.0 | 0.03636363636363636 |
| Switzerland | 5.0 | 0.09090909090909091 || | |
| --- | --- |
| age (years) | |
| n | 55 |
| mean (SD) | 52.7 (11.5) |
| median (range) | 55 (29-73) |
| duration of practice (years) | |
| n | 55 |
| mean (SD) | 24.6 (11.0) |
| median (range) | 26 (2-47) |
| duration specialized in MS (years) | |
| n | 55 |
| mean (SD) | 19.1 (9.6) |
| median (range) | 19 (0-37) |
| place of practice, n (%) | |
| city (> 100.000 residents) | 40 (72.2%) |
| town, rural area (<= 100.000 residents) | 14 (25.5%) |
| not specified | 1 (1.8%) |
| type of practice, n (%) | |
| licensed | 11 (20.0%) |
| self-employed | 10 (18.2%) |
| medical care center | 1 (1.8%) |
| clinic | 42 (76.4%) |
| outpatient | 15 (27.3%) |
| inpatient | 21 (38.2%) |
| out- and inpatient | 6 (10.9%) |
| retirement | 2 (3.6%) |
| country, n (%) | |
| Germany | 48 (87.3%) |
| Austria | 2 (3.6%) |
| Switzerland | 5 (9.1%) |
| | |
### Chart: place of practice
| Category | | |
|---|---|---|
| city
(> 100.000 residents) | 40.0 | 0.7272727272727273 |
| town, rural area
(<= 100.000 residents) | 14.0 | 0.2545454545454545 |
| not specified | 1.0 | 0.01818181818181818 |
### Chart: type of practice
| Category | | |
|---|---|---|
| licensed -
self-employed | 10.0 | 0.18181818181818182 |
| licensed -
medical care center | 1.0 | 0.01818181818181818 |
| clinic -
outpatient | 15.0 | 0.2727272727272727 |
| clinic -
inpatient | 21.0 | 0.38181818181818183 |
| clinic -
out- and inpatient | 6.0 | 0.10909090909090909 |
| retirement | 2.0 | 0.03636363636363636 |
2

## Slide 3
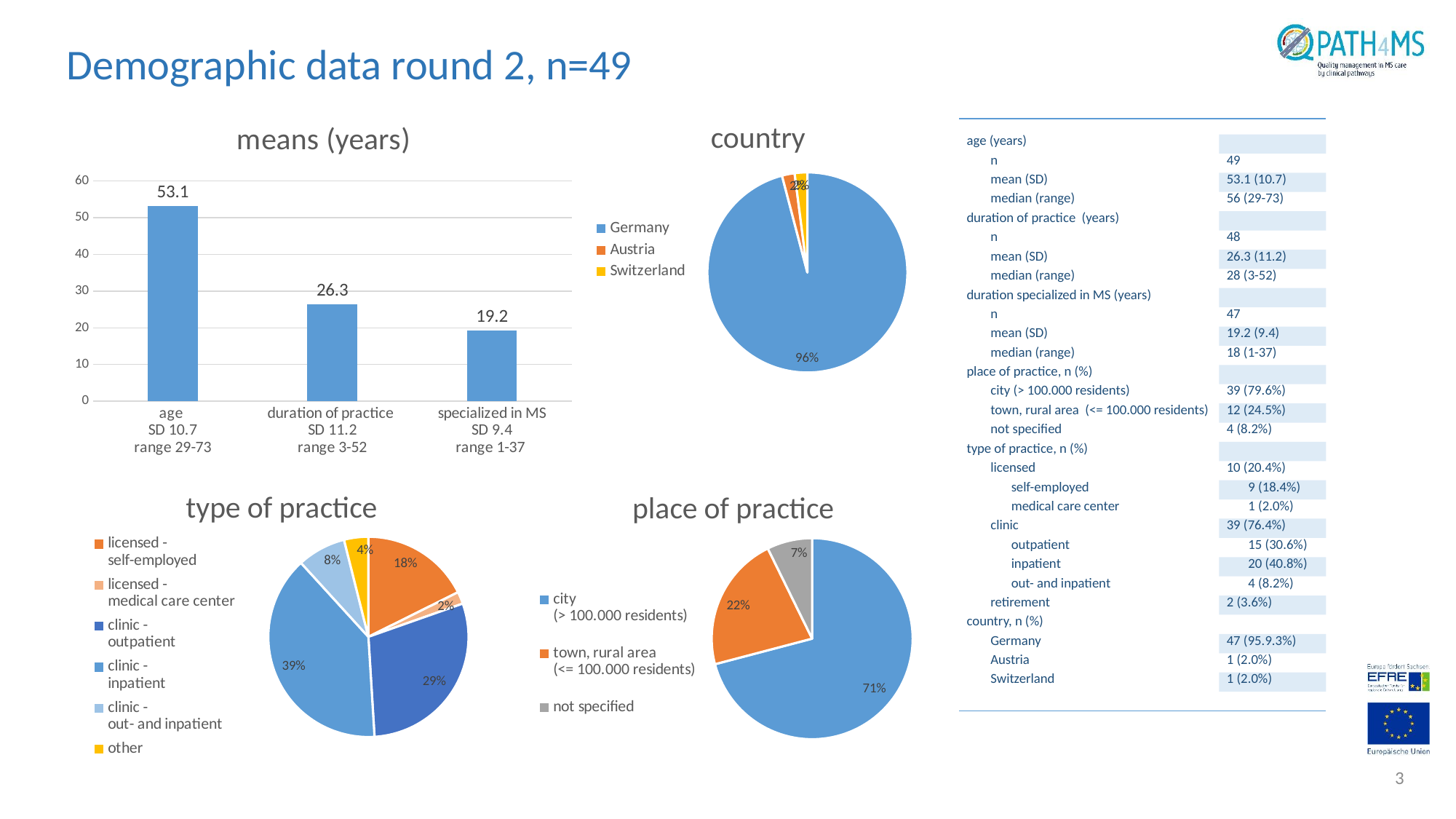

Demographic data round 2, n=49
### Chart: means (years)
| Category | |
|---|---|
| age
SD 10.7
range 29-73 | 53.1 |
| duration of practice
SD 11.2
range 3-52 | 26.3 |
| specialized in MS
SD 9.4
range 1-37 | 19.2 |
### Chart: country
| Category | | |
|---|---|---|
| Germany | 47.0 | 0.9591836734693877 |
| Austria | 1.0 | 0.02040816326530612 |
| Switzerland | 1.0 | 0.02040816326530612 || | |
| --- | --- |
| age (years) | |
| n | 49 |
| mean (SD) | 53.1 (10.7) |
| median (range) | 56 (29-73) |
| duration of practice (years) | |
| n | 48 |
| mean (SD) | 26.3 (11.2) |
| median (range) | 28 (3-52) |
| duration specialized in MS (years) | |
| n | 47 |
| mean (SD) | 19.2 (9.4) |
| median (range) | 18 (1-37) |
| place of practice, n (%) | |
| city (> 100.000 residents) | 39 (79.6%) |
| town, rural area (<= 100.000 residents) | 12 (24.5%) |
| not specified | 4 (8.2%) |
| type of practice, n (%) | |
| licensed | 10 (20.4%) |
| self-employed | 9 (18.4%) |
| medical care center | 1 (2.0%) |
| clinic | 39 (76.4%) |
| outpatient | 15 (30.6%) |
| inpatient | 20 (40.8%) |
| out- and inpatient | 4 (8.2%) |
| retirement | 2 (3.6%) |
| country, n (%) | |
| Germany | 47 (95.9.3%) |
| Austria | 1 (2.0%) |
| Switzerland | 1 (2.0%) |
| | |
### Chart: type of practice
| Category | | |
|---|---|---|
| licensed -
self-employed | 9.0 | 0.1836734693877551 |
| licensed -
medical care center | 1.0 | 0.02040816326530612 |
| clinic -
outpatient | 15.0 | 0.30612244897959184 |
| clinic -
inpatient | 20.0 | 0.40816326530612246 |
| clinic -
out- and inpatient | 4.0 | 0.08163265306122448 |
| other | 2.0 | 0.04081632653061224 |
### Chart: place of practice
| Category | | |
|---|---|---|
| city
(> 100.000 residents) | 39.0 | 0.7959183673469388 |
| town, rural area
(<= 100.000 residents) | 12.0 | 0.24489795918367346 |
| not specified | 4.0 | 0.08163265306122448 |
3
