# Supplementary material for: Consensus quality indicators for monitoring multiple sclerosis
Source: Lancet Reg Health Eur. 2024 Mar 29;40:100891. doi: 10.1016/j.lanepe.2024.100891 (PMC10998202; doi:10.1016/j.lanepe.2024.100891)
Supplement: #3 QI_Evaluation [file mmc3.pdf]

Supplement #3

evaluation

Results of evaluation: criterion 1

Does the QI makes sense?

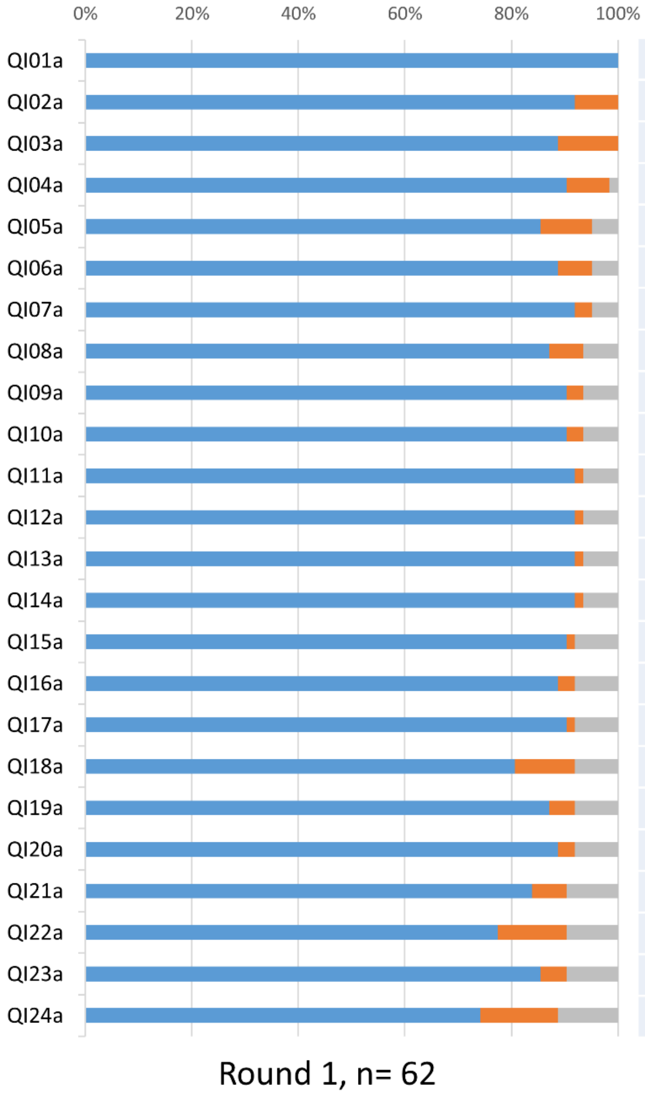

Monitoring Work-Up
Symptoms Checklist
Screening – differential diagnostics – therapy(options)
Checklist FS visual
Checklist FS brainstem
Checklist FS pyramidal
Checklist FS cerebellar
Checklist FS sensory
Checklist FS bladder
Checklist FS bowel
Checklist FS sexual dysfunction
Checklist FS cerebral mental neuropsychological
Checklist FS ambulation and mobility
Comorbidities und medication
Individual care
Checklist social medical
Checklist lifestyle
Checklist care
Standardized neurological examination and evaluation
Standardized MRI-diagnostics: cerebral
Standardized MRI-diagnostics: spinal
Additional assessment
Evaluation with recommended action
Frequency Monitoring Work-Up

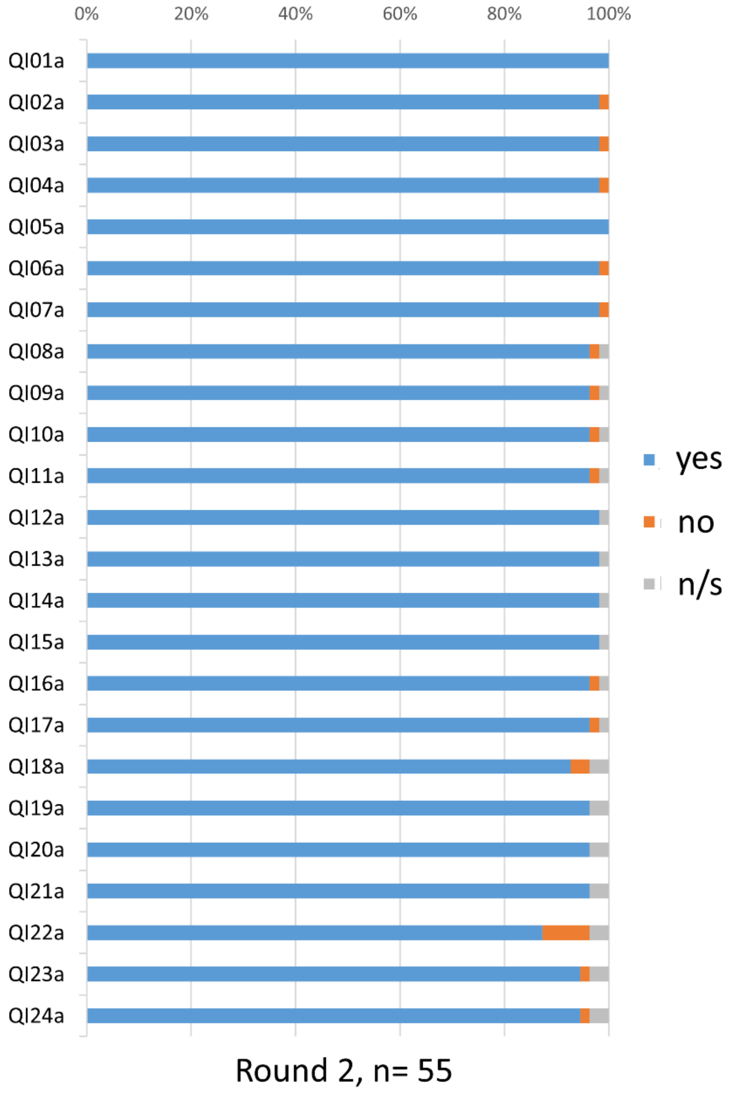

Results of evaluation: criterion 2

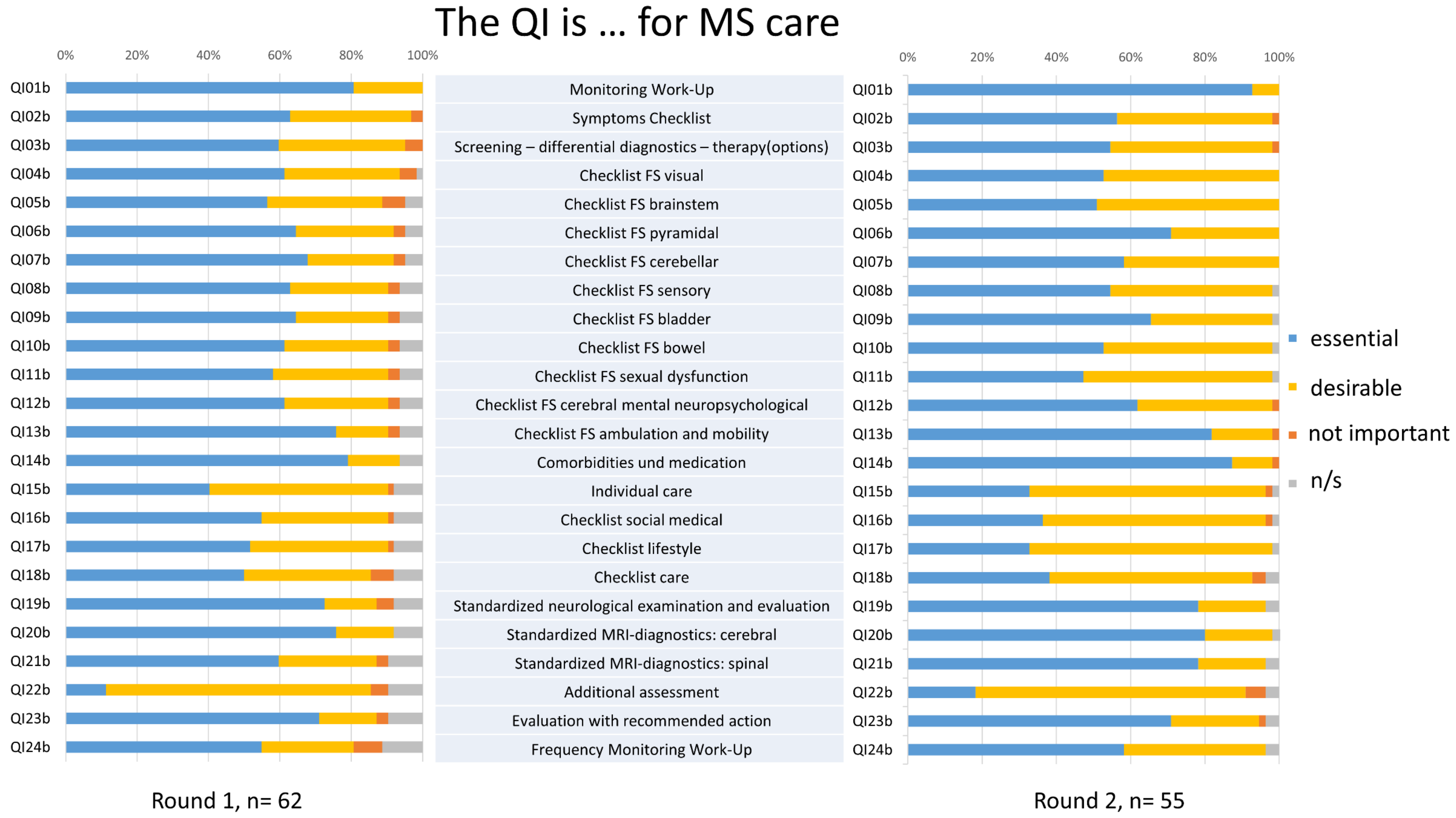

PATH4MS
Quality management in MS care by clinical pathways

Results of evaluation: criterion 3

Should this QI be communicated to the patient?

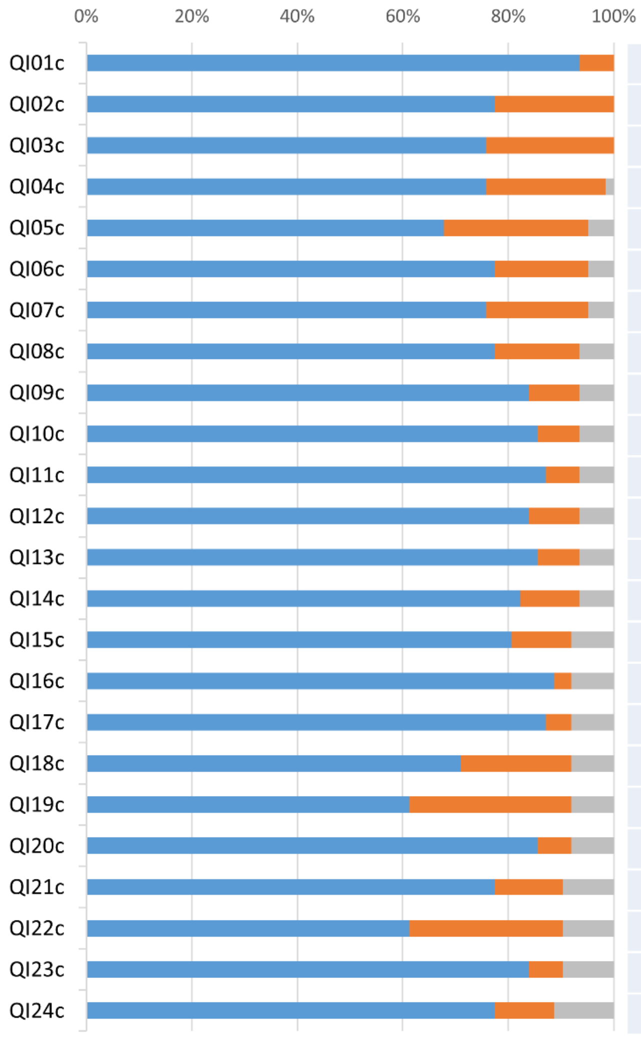

Round 1, n= 62

Monitoring Work-Up
Symptoms Checklist
Screening – differential diagnostics – therapy(options)
Checklist FS visual
Checklist FS brainstem
Checklist FS pyramidal
Checklist FS cerebellar
Checklist FS sensory
Checklist FS bladder
Checklist FS bowel
Checklist FS sexual dysfunction
Checklist FS cerebral mental neuropsychological
Checklist FS ambulation and mobility
Comorbidities und medication
Individual care
Checklist social medical
Checklist lifestyle
Checklist care
Standardized neurological examination and evaluation
Standardized MRI-diagnostics: cerebral
Standardized MRI-diagnostics: spinal
Additional assessment
Evaluation with recommended action
Frequency Monitoring Work-Up

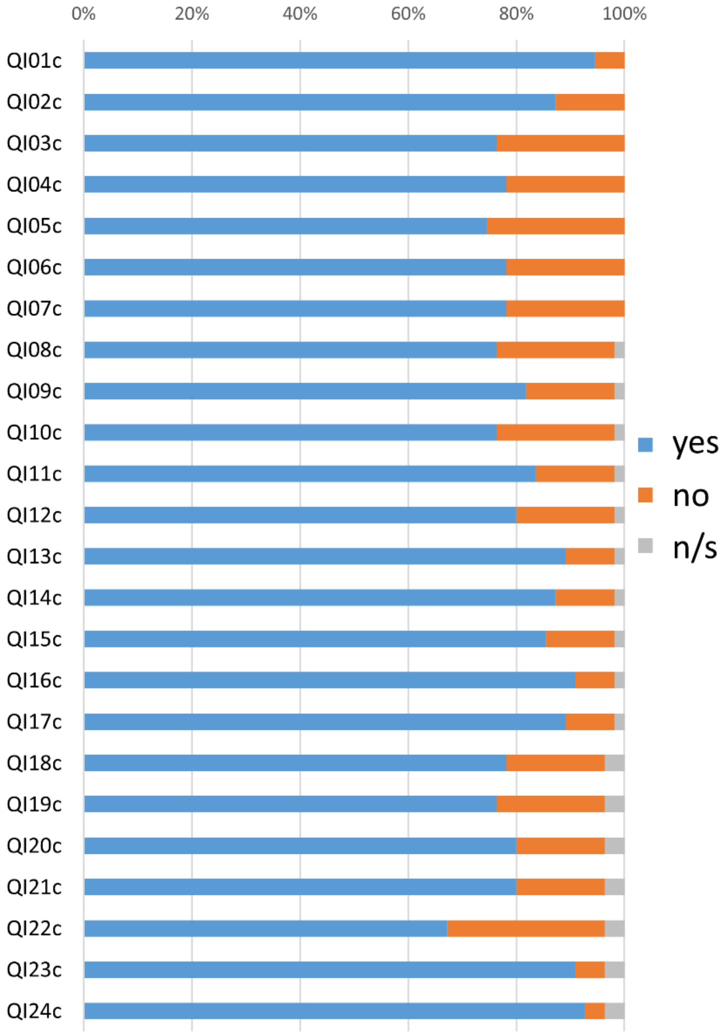

Round 2, n= 55

Europa fördert Sachsen. EFRE
Europäische Union
